# Biases during DNA extraction affect characterization of the microbiota associated with larvae of the Pacific white shrimp, *Litopenaeus vannamei*

**DOI:** 10.7717/peerj.5257

**Published:** 2018-07-16

**Authors:** Ming Xue, Liyou Wu, Yaoyao He, Huafang Liang, Chongqing Wen

**Affiliations:** 1 Fisheries College, Guangdong Ocean University, Zhanjiang, Guangdong, China; 2 Institute for Environmental Genomics, and Department of Microbiology and Plant Biology, University of Oklahoma, Norman, OK, USA

**Keywords:** *Litopenaeus vannamei* larvae, DNA extraction, Commercial kit, Bacterial community, High-throughput sequencing

## Abstract

For in-depth characterization of the microbiota associated with shrimp larvae, careful selection of DNA isolation procedure is paramount for avoiding biases introduced in community profiling. Four E.Z.N.A.™ DNA extraction kits, i.e., Bacterial, Mollusc, Stool, and Tissue DNA Kits, abbreviated as Ba, Mo, St, and Ti, respectively, were initially evaluated with zoea 2 (Z2) larvae of the Pacific white shrimp (*Litopenaeus vannamei*) by 16S amplicon sequencing on a Illumina MiSeq platform. Further characterization of additional larval samples, specifically nauplii 5 (N5), mysis 1 (M1), and postlarvae 1 (P1), was performed with Ba and St kits to examine the changing microbiota profile during shrimp hatchery period. The results from the Z2 samples showed that DNA yields from the four kits varied significantly (*P* < 0.05), whereas no significant differences were detected in the α-diversity metrics of the microbiota. By contrast, the St kit, with the lowest DNA yield and quality, successfully recovered DNA from Gram-positive and gut-associated bacterial groups, whereas the Ba kit, which showed maximal microbiota similarity with the Mo kit, manifested the best reproducibility. Notably, significant differences were observed in relative abundances of most dominant taxa when comparing results from the Ba and St kits on Z2, M1, and P1 samples. In addition, the bacterial community identified shifted markedly with larval development regardless of the DNA extraction kits. The DNA recovery biases arising from the larval microbiota could be due to different protocols for cell lysis and purification. Therefore, combined application of different DNA extraction methods may facilitate identification of some biologically important groups owing to their complementary effects. This approach should receive adequate attention for a thorough understanding of the larvae-associated microbiota of the penaeid shrimp.

## Introduction

The Pacific white shrimp, *Litopenaeus vannamei* (Boone), one of the best-known aquaculture species globally, experiences continual metamorphosis during its hatchery period. As is well known, the entire cycle of *L. vannamei* larviculture is controlled under condition of indoor intensive environment with high stocking density, frequent diet feeding and low water exchange. The reliable production of high-quality shrimp larvae cannot be guaranteed due to high mortality rates (Vandenberghe et al., 1999; Kumar et al., 2017). These failures may be partly ascribed to diseases that result from dysbiosis in the shrimp larval microbiota, because symbiotic microorganisms contribute greatly to larval health (Zheng et al., 2016; Xiong et al., 2017b).

To date, cultivation-based and conventional molecular methods have been used to characterize the microbial community associated with *L. vannamei* larvae (Vandenberghe et al., 1999; Garcia & Olmos, 2007; Pangastuti et al., 2010; Zheng et al., 2016). In comparison with the traditional microbial techniques, high-throughput sequencing is increasingly being adopted as its merit to reveal vast microbial diversity of environmental samples (Wu et al., 2015). For example, Huang et al. (2016) and Zeng et al. (2017) compared changes in the intestinal microbiota of *L. vannamei* at different culture stages, whereas Xiong, Zhu & Zhang (2014) and Xiong et al. (2017a; 2017b) analyzed differences in the intestinal microbiota of *L. vannamei* in various health or growth states. However, these studies have mainly focused on juvenile and adult shrimp. More recently, Zheng et al. (2017) reported the bacterial community composition associated with healthy and diseased *L. vannamei* larvae as well as with larviculture water.

Although the exploration of microbial diversity is no longer limited by the bottlenecks due to sequencing, DNA fidelity is of great concern. Besides, biases during DNA extraction, which have been documented by diverse fingerprinting or high-throughput sequencing methods of various samples, may affect downstream analysis (Salonen et al., 2010; Maukonen, Simões & Saarela, 2012; Yuan et al., 2012; Guo & Zhang, 2013; Kennedy et al., 2014b; Rubin et al., 2014; Larsen, Mohammed & Arias, 2015). In fact, many commercial DNA extraction kits have been designed based on the physical properties of targeted samples, such as stool, soil, mollusks, and animal tissue. Nevertheless, no commercial DNA extraction kit is specifically designed for crustaceans or their larvae. Shrimp larvae, which are too small to be dissected individually, are surrounded by an exoskeleton similar to that of insects and other hard-bodied arthropods (Rubin et al., 2014). This situation offers challenges for extracting the DNA from their endosymbiotic microorganisms (Ammazzalorso et al., 2015). Accordingly, a suitable DNA extraction method is necessary for high-throughput sequence analysis of the shrimp larvae-microbiota.

In general, most of commercial kits differ in cell lysis and DNA purification procedures, which have been regarded as two main factors influencing microbial DNA extracts (Yuan et al., 2012; Guo & Zhang, 2013). In this study, four commercial kits were initially screened based on DNA yield and quality, diversity metrics, reproducibility, and the ability to recover Gram-positive bacteria associated with *L. vannamei* zoea larvae. Then, two superior kits among these four were further evaluated to verify their suitability, with another three larval samples of nauplius, mysis, and postlarvae. These results will be conducive to optimization of DNA extraction protocols and characterization of the marine-crustacean-larvae-associated microbiota. Our data will also promote investigation of the relations between shrimp larvae and their symbiotic and/or epiphytic microbes.

## Materials and Methods

### Larvae rearing and sample collection

*L. vannamei* larvae were reared in an indoor larviculture pond at a commercial hatchery located on Donghai Island, Guangdong Province, China. The protocols of seawater disinfection, larvae stocking, and diet feeding were described previously by Xue et al. (2017). During the nursery period, seawater was constantly aerated, with temperature, salinity, and pH maintained in ranges of 28.5–32.4 °C, 28.0–31.5%, and 7.69–8.35, respectively. In addition, shrimp larvae were active and showed no signs of disease upon visual inspection throughout the larviculture period.

Larval samples were obtained at four time points, corresponding to the larval stages of nauplii 5 (N5), zoea 2 (Z2), mysis 1 (M1), and postlarvae 1 (P1) at 12 h, 2, 4, and 8 days after larvae inoculation, respectively. For sampling, a total of six L of rearing water (1.5 L aliquots from four locations of the pond), was collected by a handled sterile beaker and loaded into aseptic polyethylene bags. After filtration of seawater through a 300-mesh gauze, larvae were obtained and rinsed three times with sterile seawater, then stored at −20 °C until DNA extraction within 1 week.

### DNA extraction

Four E.Z.N.A. DNA extraction kits (OMEGA Bio-Tek Inc., Norcross, GA, USA), designed separately for bacteria, mollusks, stool, and animal tissues, were initially evaluated for their extraction efficiencies toward Z2 larvae. The abbreviations and main properties of these kits are summarized in Table 1.

**Table 1:** Characteristics of the four DNA extraction kits.

DNA extraction kit	Abbreviation	Cell lysis	DNA purification
Bacterial DNA kit (D3350)	Ba	E1, E2, M	Silica column
Mollusc DNA kit (D3373)	Mo	E2, C	Silica column
Stool DNA kit (D4015)	St	E2, T, M	Silica column
			Adsorbing inhibitors with HTR reagent
Tissue DNA kit (D3996)	Ti	E3	Silica column

**Note:**

M, mechanical (bead beating); T, temperature (thermal shock); E, enzymatic (E1: lysozyme, E2: proteinase K, E3: OB protease); C, chemical (chloroform: isoamyl alcohol).

Prior to DNA extraction, identical tissue homogenization was undertaken. Approximately 200 Z2 larvae were homogenized in a small amount of cold TE (Tris-EDTA) buffer on ice by means of a hand-held tissue homogenizer, and were mixed thoroughly to a final volume of five mL. Twelve 300 μL aliquots of the homogenates were separately transferred into 1.5 mL Eppendorf tubes and centrifuged at 4 °C for 5 min at 12,000 × g, and the supernatant was discarded. DNA was extracted from three pellets with each kit as per the manufacturer’s instructions, and the extracted DNA was dissolved in 100 μL of TE buffer and stored at −20 °C.

The cell lysis procedures for these kits are as follows: (1) Ba kit: robust vortexing of the sample with glass powder after incubation with lysozyme at 30 °C for 30 min, then incubation with proteinase K at 55 °C for 1 h. (2) Mo kit: extraction of the sample with chloroform–isoamyl alcohol in the ratio of 24:1, after proteinase K hydrolysis at 60 °C for 1 h. (3) St kit: vortexing the sample at maximum speed for 5 min with glass beads and proteinase K incubation at 70 °C for 10 min, followed by 90 °C for 5 min, and on ice for 2 min. (4) Ti kit: hydrolysis of the sample with OB proteinase at 55 °C for 1 h. For DNA purification, spin filters were commonly employed for all the kits, except that there was an additional step for adsorbing inhibitors with a matrix in the St kit. The sample sizes of N5, M1, and P1, subjected to DNA extraction with kits Ba and St, were 300, 100, and 30 larvae, respectively. Unless stated otherwise, all extraction procedures for these three samples, in triplicate for each kit, were the same as those applied to the Z2 samples.

### DNA quantification

The DNA extracts were qualified and quantified on a NanoDrop ND-1000 spectrophotometer (NanoDrop^®^ Technologies, Wilmington, DE, USA). In addition, DNA concentration was also quantified by PicoGreen (Promega, Sunnyvale, CA, USA) (Ahn, Costa & Emanuel, 1996) on a FLUOstar OPTIMA plate reader (BMG Labtech, Jena, Germany), and the template DNA was diluted to 2 ng μL^−1^ with nuclease-free water (Ambion, Austin, TX, USA).

### PCR amplification and MiSeq sequencing

Primers 515F (5′-GTGCCAGCMGCCGCGGTAA-3′) and 806R (5′-GGACTACHVGGGTWTCTAAT-3′) (Caporaso et al., 2011), were used to amplify the V4 region of the 16S rRNA genes in accordance with previously described methods (Wu et al., 2015; Wen et al., 2017). Briefly, polymerase chain reaction (PCR) amplification was carried out in triplicate in a total volume of 25 μL, consisting of 1 × AccuPrime buffer II, 0.5 U of AccuPrime™ Taq (Invitrogen, Carlsbad, CA, USA), 0.4 mM each primer, and 10 ng of template DNA. Amplification conditions started with an initial denaturation step for 3 min at 94 °C, followed by 28 cycles of 94 °C for 20 s, 53 °C for 25 s, and elongation at 68 °C for 45 s. A final extension for 10 min at 68 °C was performed. Positive PCR products were confirmed by agarose gel electrophoresis, and products from triplicate reactions were combined for quantification by PicoGreen.

All 12 Z2 samples, each with 400 ng of PCR product, were pooled and purified using the QIAquick Gel Extraction Kit (Qiagen, Valencia, CA, USA), followed by re-quantification with PicoGreen. The resulting library was sequenced (as the first run) at the Institute for Environmental Genomics at the University of Oklahoma on the MiSeq platform (Illumina, San Diego, CA, USA) with the 2 × 250 bp kit. The library preparation and sequencing of N5, M1, and P1, which were extracted with the Ba and St kits, were the same as those for Z2, but were performed in the second sequencing run.

### Data processing

Quality filtering and processing of the sequences was performed on the Galaxy bioinformatics pipeline (http://zhoulab5.rccc.ou.edu:8080/root), as described elsewhere (Wu et al., 2015; Wen et al., 2017), with modifications including assigning the sequences to the appropriate samples without a mismatch with their barcodes. Paired-end reads of sufficient length (at least 50 base overlap between forward and reverse reads) were merged into full-length sequences by FLASH v.1.2.8 (Magoč & Salzberg, 2011). Sequencing data reported in this paper were deposited in the Genome Sequence Archive in BIG Data Center, Beijing Institute of Genomics, CAS, under accession number CRA000198 that is publicly accessible at http://bigd.big.ac.cn/. An operational taxonomic unit (OTU) table without singletons was generated by UPARSE (Edgar, 2013) at a 97% similarity level. Taxonomic annotations were assigned to each OTU’s representative with the most abundant sequence by means of the RDP Classifier (Wang et al., 2007). A few taxa were revised using the EzBioCloud’s Identify Service (http://www.ezbiocloud.net/identify). Prior to downstream analysis, all the samples were resampled to the same sequencing depth, based on the sample with the lowest sequence number.

Alpha diversity analysis was performed using the Shannon diversity index, Pielou’s evenness index, and the Chao1 value, and Good’s coverage of Z2 was evaluated as described by Zhou et al. (2011). The similarity indices of Jaccard, Sorensen, and Bray–Curtis were chosen to compare the technical reproducibility within a kit or between the kits as described by Wen et al. (2017). Detrended correspondence analysis (DCA), based on OTU data, was performed in software CANOCO 4.5 to visualize the ordination relation of Z2 larval microbiota extracted with the four kinds of kits. Dominant taxa at phylum (or class for Proteobacteria), family, and OTU-level were defined as those having ≥1% relative abundance at least in one of the four kits. A heat map, constructed by the gplots package with software R 3.2.4 (www.R-project.org), was used to visualize the cluster relations of dominant OTU-based communities of the four larval stages.

### Statistical analysis

The relative abundance percentages of various taxa were presented as mean ± SD and were transformed with the arcsine of the square root for statistical analysis. All the data were tested for normality prior to analysis. One-way analysis of variance was carried out to test the significance (*P* < 0.05) of DNA quantification and α-diversity indices among the four kits, followed by Duncan’s multiple range test, when a significant difference was detected. The significance of dominant taxa at family and OTU levels between the Ba and St kits was analyzed by the wilcoxon rank-sum test. All the analyses were performed in software R 3.2.4, and package agricolae was employed for multiple range test.

## Results

### DNA qualification and quantification

The DNA quality and yield from Z2 larvae extracted by the four kits are shown in Table 2. The *OD*_260_/*OD*_280_ ratios were similar in extracts from the kits Ba, Mo, and Ti, which approached the optimal level of 1.8, whereas the St kit had a significantly lower ratio (*P* < 0.05), indicating the presence of more protein and/or other impurities. DNA concentration varied significantly (*P* < 0.05) in the ranges of 3.73–27.2 and 2.73–26.23 ng μL^−1^ when measured by means of NanoDrop and PicoGreen, respectively. Overall, the Mo kit had the highest DNA yields, whereas the St kit presented significantly lower yields (*P* < 0.05).

**Table 2:** DNA yield and quality of Z2 samples extracted with four kits.

Kits	*OD*_260_/*OD*_280_	DNA concentration (ng/μL)	
		NanoDrop	PicoGreen
Ba	1.64 ± 0.07^a^	21.01 ± 3.87^b^	22.01 ± 3.61^a^
Mo	1.65 ± 0.05^a^	27.20 ± 3.99^a^	26.23 ± 3.14^a^
St	1.16 ± 0.14^b^	3.73 ± 0.35^c^	2.73 ± 0.20^b^
Ti	1.60 ± 0.15^a^	21.33 ± 0.65^b^	5.47 ± 1.02^b^

**Note:**

Means with different superscript letters in the same column are significantly different (*P* < 0.05, *n* = 3).

### OTU-based α-diversity analysis of Z2 samples

In the first sequencing run for 12 samples of Z2 larvae, the triplicate extracts of kits Ba, Mo, St, and Ti generated 67,637 ± 19,490, 55,970 ± 1,519, 43,953 ± 5,499, and 50,121 ± 5,608 sequence reads, respectively, after quality control and OTU filtering (Table S1). The lowest sequencing depth 37,722 was seen in sample StZ2_3. The OTU table for the 12 samples was resampled at the same depth of 37,722 for subsequent analysis. On the basis of the 97% sequence similarity, the St kit yielded the highest OTU number, whereas no statistical differences in OTU counts were detected among the four kits (Table 3). Similarly, all α-diversity metrics, including the Shannon diversity index, Pielou evenness index, and richness index (Chao l value) revealed no significant differences among the kits.

**Table 3:** OTU-based α-diversity of microbiota of Z2 samples subjected to extraction with four kits.

Kits	OTU number	Chao1 value	Coverage (%)	Shannon diversity index	Pielou’s evenness index
Ba	217 ± 3^a^	261 ± 15^a^	99.90 ± 0.01^a^	2.96 ± 0.06^a^	0.55 ± 0.01^a^
Mo	273 ± 170^a^	335 ± 216^a^	99.85 ± 0.13^a^	2.93 ± 0.34^a^	0.53 ± 0.01^a^
St	352 ± 123^a^	405 ± 103^a^	99.83 ± 0.03^a^	3.11 ± 0.10^a^	0.54 ± 0.02^a^
Ti	263 ± 23^a^	310 ± 20^a^	99.88 ± 0.01^a^	2.96 ± 0.17^a^	0.53 ± 0.02^a^

**Note:**

Means with the same superscript letters in the same column are not significantly different (*P* > 0.05, *n* = 3).

### Microbiota similarities within and between kits

**Figure 1:**
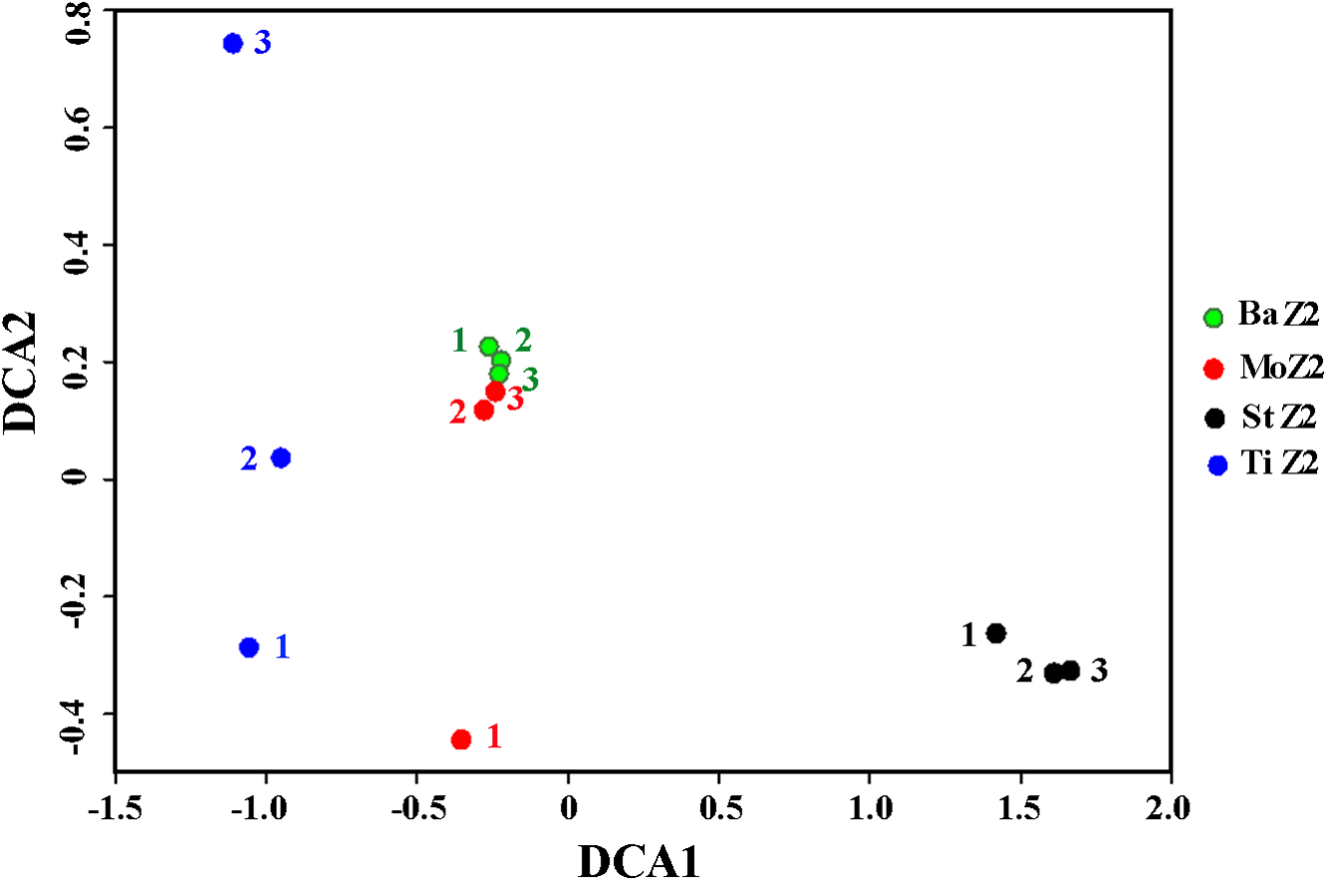
Detrended correspondence analysis of the Z2 larval microbiota from DNA extracted with four kits. BaZ2, MoZ2, StZ2, and TiZ2 indicate microbial DNA extracted from Z2 larvae with kits Ba, Mo, St, and Ti, respectively; numbers 1, 2, and 3 indicate technical replicates of each kit.

Table 4 shows the similarity indices among replicates within one kit or between kits, according to OTU data. Within a kit, the Ba kit exhibited the best similarity, the St kit took the second place, whereas kits Mo and Ti appeared relatively worse. This result was further visualized by DCA plot of the bacterial community differentiation among the four kits (Fig. 1), i.e., the three technical replicates of Ba clustered together tightly, and the corresponding ones of St were also close to each other, whereas one replicate of Mo deviated greatly from the other two, and the degree of dispersion of three Ti replicates was even higher. Among these kits, the maximal community similarity was noticed between kits Ba and Mo with regard to all three metrics, especially according to the Bray–Curtis index, which was significantly higher than that in the other pairwise kit comparisons (Table 4). Thus, the Ba and St kits were more reliable in terms of DNA extraction, showing better reproducibility.

**Table 4:** Similarity indices among replicates within a kit and between kits.

Level	Data size*	Similarity index		
		Jaccard	Sorensen	Bray–Curtis
Ba	3	0.4560 ± 0.0115^a^	0.6263 ± 0.0110^a^	0.9023 ± 0.0090^a^
Mo	3	0.2837 ± 0.0794^b^	0.4377 ± 0.0943^b^	0.8553 ± 0.0558^ab^
St	3	0.3767 ± 0.0688^ab^	0.5447 ± 0.0706^ab^	0.9022 ± 0.0196^a^
Ti	3	0.3550 ± 0.236^ab^	0.5240 ± 0.0256^ab^	0.7960 ± 0.7960^b^
Ba–Mo	9	0.3527 ± 0.0661^ab^	0.5181 ± 0.0751^ab^	0.8562 ± 0.0377^a^
Ba–St	9	0.3167 ± 0.0566^bc^	0.4783 ± 0.0667^bc^	0.3190 ± 0.0448^c^
Ba–Ti	9	0.3704 ± 0.0252^a^	0.5401 ± 0.0267^a^	0.5536 ± 0.0456^b^
Mo–St	9	0.2787 ± 0.0491^c^	0.4334 ± 0.0601^c^	0.3084 ± 0.0456^c^
Mo–Ti	9	0.3136 ± 0.0388^bc^	0.4761 ± 0.0450^bc^	0.5489 ± 0.0448^b^
St–Ti	9	0.3050 ± 0.0454^bc^	0.4659 ± 0.0545^bc^	0.2996 ± 0.0384^c^

**Notes:**

Means with different superscripts in the same column are significantly different (*P* < 0.05).

* Indicates the number of replicates of each kit or of data points of the pairwise comparisons within the technical replicates of any two kits.

### Dominant bacterial phyla/classes and families of Z2 larvae

**Figure 2:**
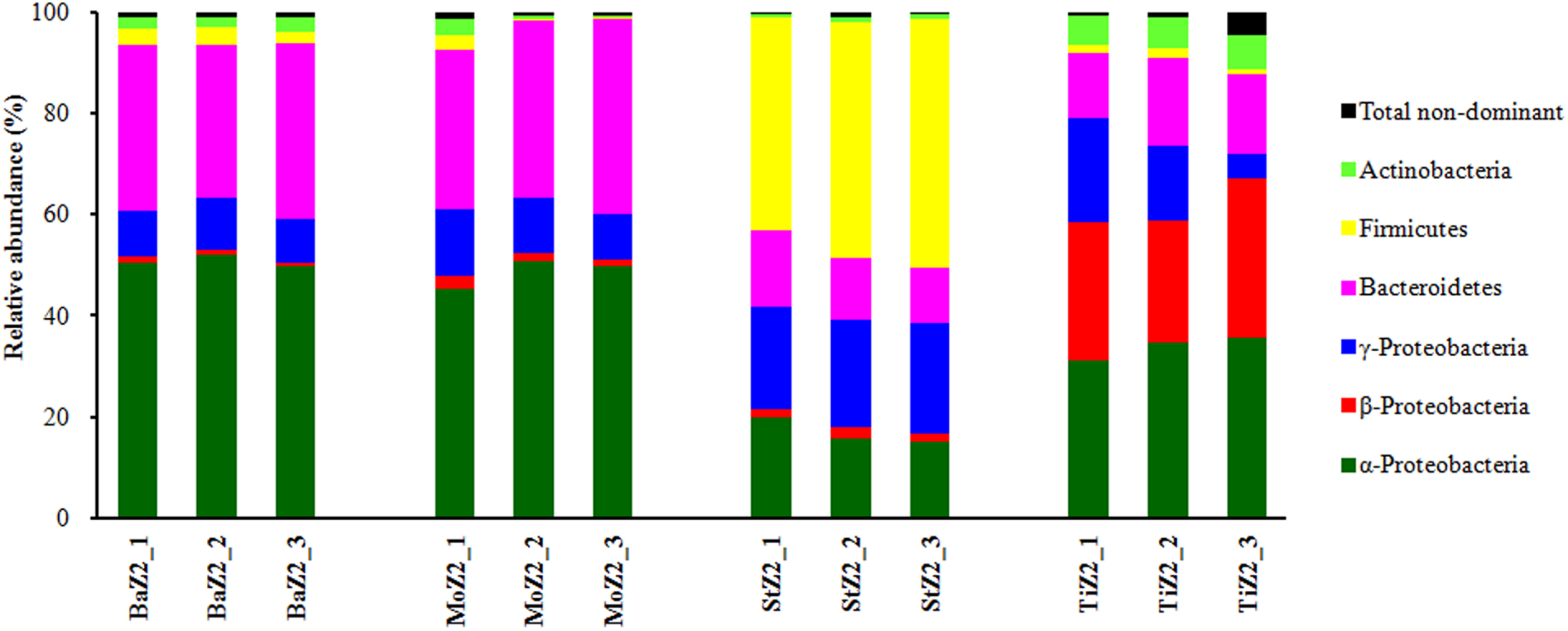
Relative abundance of dominant bacterial phyla or classes associated with Z2 larvae extracted with four DNA isolation kits. BaZ2, MoZ2, StZ2, and TiZ2 indicate microbial DNA extracted from Z2 larvae with kits Ba, Mo, St, and Ti, respectively; numbers 1, 2, and 3 indicate technical replicates of each kit.

Dominant phyla or classes of Z2 larvae-associated microbiota are depicted in Fig. 2. Comparison of the relative abundances among these six phyla/classes revealed significant differences (*P* < 0.05) among the four kits. Nevertheless, no significant difference was detected in each Ba and Mo pair with respect to the most frequent taxa. Moreover, the phylum Firmicutes, comprising Gram-positive bacteria, was predominant in St extracts with relative abundance up to 46.03%, which was significantly higher than that of the other three kits (1.22–2.97%).

The relative abundance levels of 14 dominant bacterial families associated with Z2 larvae extracted with the four kits are shown in Table S2. With kits Ba and Mo, 11 families had comparable proportions, except for Leuconostocaceae, Streptococcaceae, and Nocardioidaceae, which manifested significantly higher (*P* < 0.05) levels in Ba extracts. When kits Ba and St being compared, seven out of 14 families (Rhodobacteraceae, Alteromonadaceae, Bacillaceae, Flavobacteriaceae, Cyclobacteriaceae, Nocardioidaceae, and Unclassified OTU19) showed significantly higher (*P* < 0.05) relative abundances in the Ba kit, whereas the proportions of the other six families (Oxalobacteraceae, Enterobacteriaceae, Moraxellaceae, Listeriaceae, Leuconostocaceae, and Streptococcaceae) were significantly higher in the St kit. The kits Ba and St were next utilized for DNA isolation from other larval samples. This is because kit Ba yielded a microbiota similar to that of Mo, but had the best reproducibility, and St had good reproducibility and the ability to successfully extract Gram-positive bacteria. Kit Ti was disused because of its poor repeatability.

### Microbiota profiling during four larval stages

**Figure 3:**
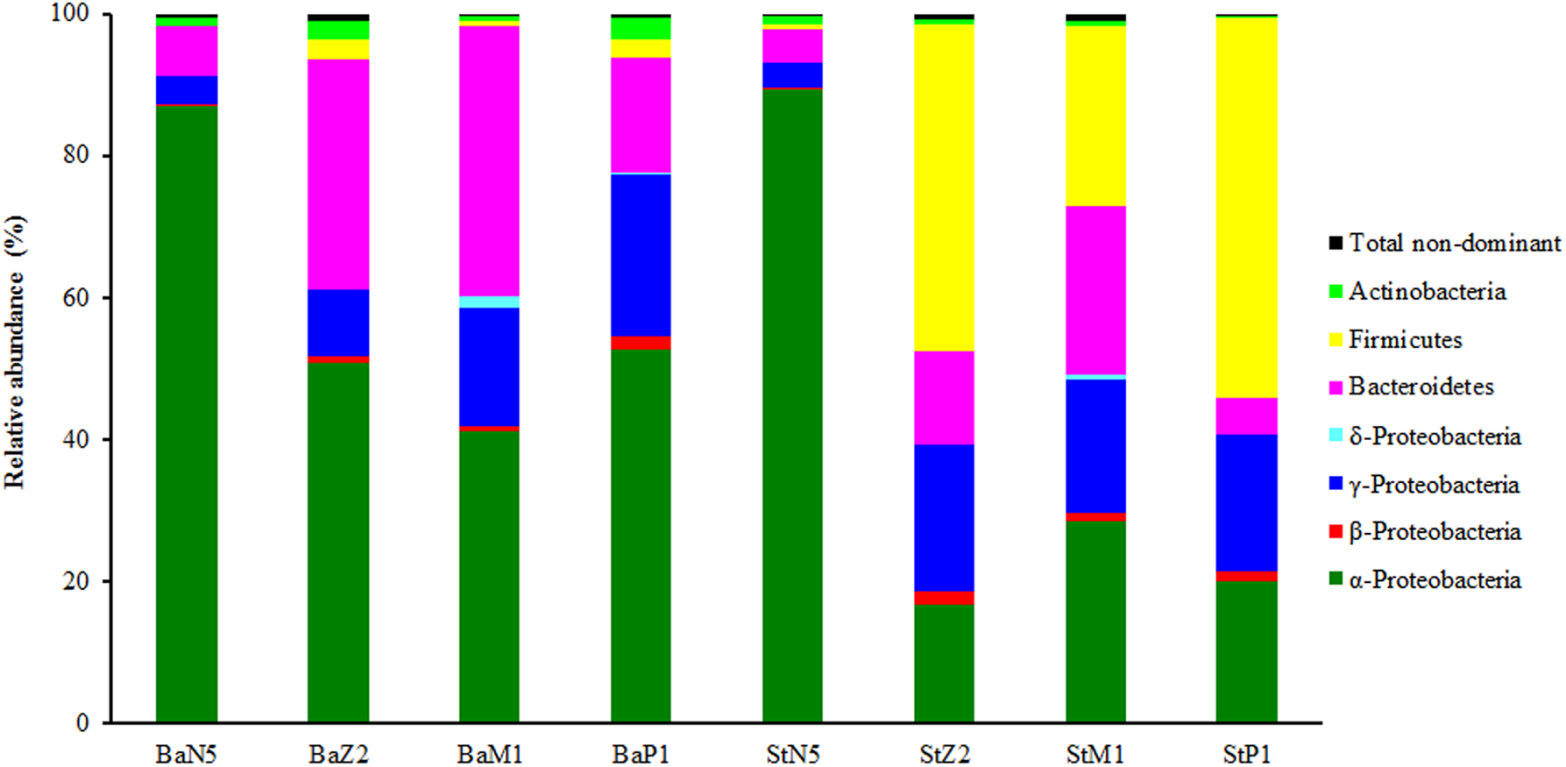
Composition and abundance of dominant bacterial phyla or classes associated with *L. vannamei* larvae at stages of N5, Z2, M1, and P1 (*n* = 3). BaN5, BaZ2, BaM1, and BaP1 indicate microbial DNA extracted with the Ba kit from larval samples of stages N5, Z2, M1, and P1, respectively. This is similar for the St kit.

In the second sequencing run for N5, M1, and P1 samples, the sequence reads from extracts of kit Ba were 23,671 ± 4,100, 84,756 ± 31,669, and 18,990 ± 738, respectively, and the corresponding ones from kit St were 27,788 ± 1,704, 55,366 ± 1,117, and 19,680 ± 1,309 respectively, after quality control and OTU filtering (Table S1). On the basis of the minimal sequence number of sample BaP1_3 (Table S1), the OTU table for four larval-stage samples with kits Ba and St was resampled at the same depth of 18,180 for microbiota analysis. Figure 3 shows the relative abundance levels of dominant bacterial phyla/classes across different larval stages. Overall, there was a highly dynamic microbiota structure during larval development, where α-Proteobacteria, γ-Proteobacteria, and Bacteroidetes were the most dominant groups. Notably, Firmicutes was predominant in the later three larval stages of St extracts. By contrast, the microbiota structure of N5 larvae differed markedly from the other stages, with α-Proteobacteria as the dominant group for both kits. From Z2 to P1, α-Proteobacteria still dominated in Ba extracts, while the relative abundance of γ-Proteobacteria continuously increased. Additionally, when larvae developed from N5 to M1, Bacteroidetes increased progressively in abundance as the second dominant group, slightly dropping in abundance at P1. Therefore, these composition analyses indicated variation in the efficiency of recovery of the larval microbiota with the Ba and St kits.

**Figure 4:**
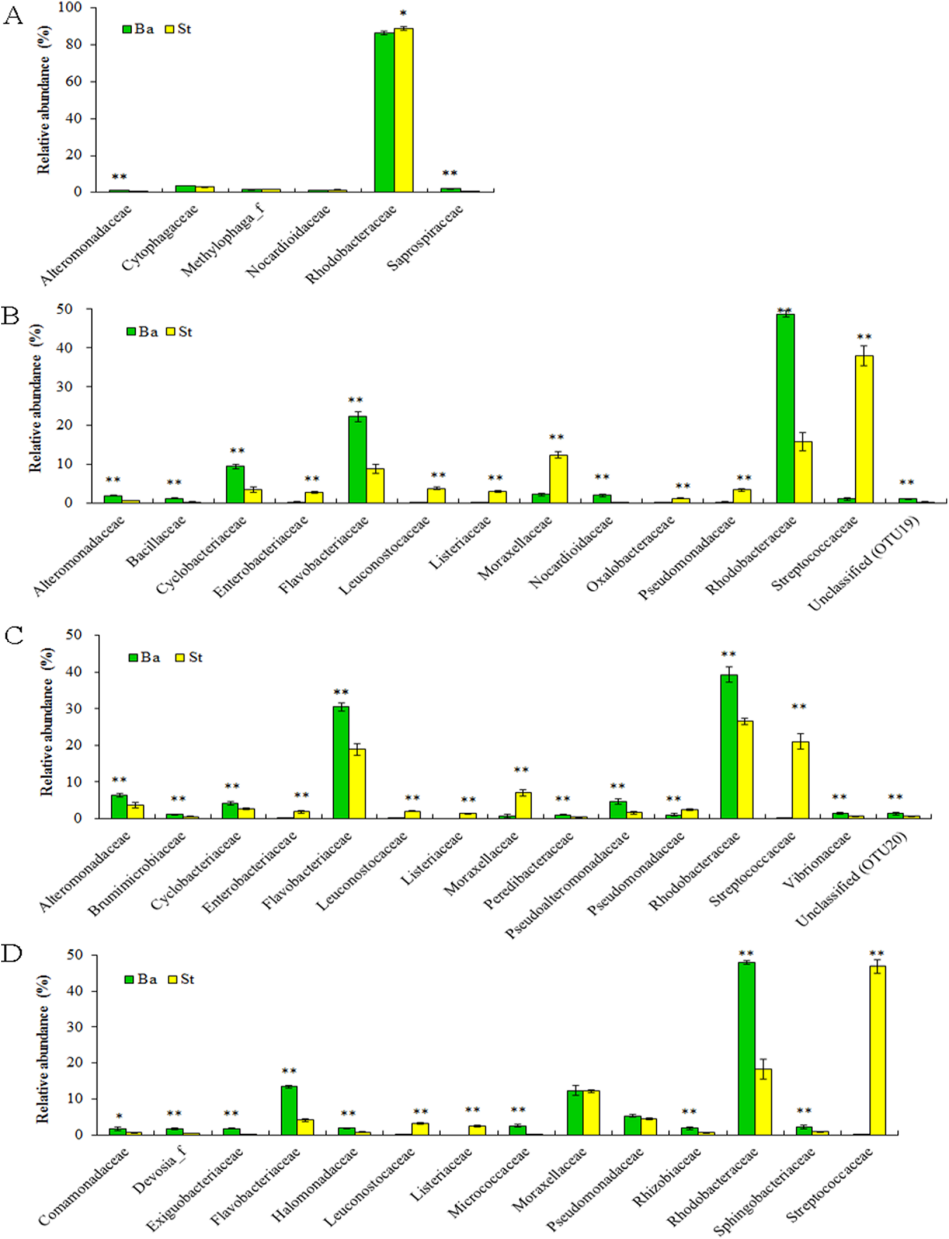
Dominant bacterial families associated with the *L. vannamei* larvae at stages N5 (A), Z2 (B), M1 (C), and P1 (D). *, ** on the columns denote significant difference with *P* < 0.05 and *P* < 0.01, respectively, between the Ba and St kits.

Dominant families and their statistically significant differences in relative abundance between the Ba and St kits for four larval-stage samples are shown in Fig. 4. For all the samples, Rhodobacteraceae dominated mostly in both extracts. Flavobacteriaceae and Streptococcaceae were the subdominant groups for kits Ba and St respectively for the later three samples. A total of three, 14, 15, and 12 families manifested significant differences (*P* < 0.05) at N5, Z2, M1, and P1 stages, respectively. For N5, the relative abundances of Alteromonadaceae and Saprospiraceae were significantly higher (*P* < 0.01) with the Ba kit than with the St kit. Nonetheless, Rhodobacteraceae predominated in both Ba and St kits, but was significantly higher in abundance (*P* < 0.05) in the latter. At the following larval stages, with the exception of Moraxellaceae and Pseudomonadaceae at P1, all the dominant families showed significant differences between Ba and St kits. Three families, including Streptococcaceae, Leuconostocaceae, and Listeriaceae, all affiliated with Firmicutes, revealed total proportions of 44.81%, 24.54%, and 52.52% at Z2, M1, and P1 stages, respectively, with the St kit. In contrast, these values with the Ba kit were as low as 0.07–1.10%. Furthermore, Enterobacteriaceae with the St kit showed relatively high abundance levels of 2.81%, 1.86%, and 0.88% at stages Z2, M1, and P1, compared to 0.30%, 0.21%, and 0.12% with the Ba kit, respectively. These results confirmed that the St kit had robust capacity for recovery of Gram-positive bacteria and some enteric bacteria.

**Figure 5:**
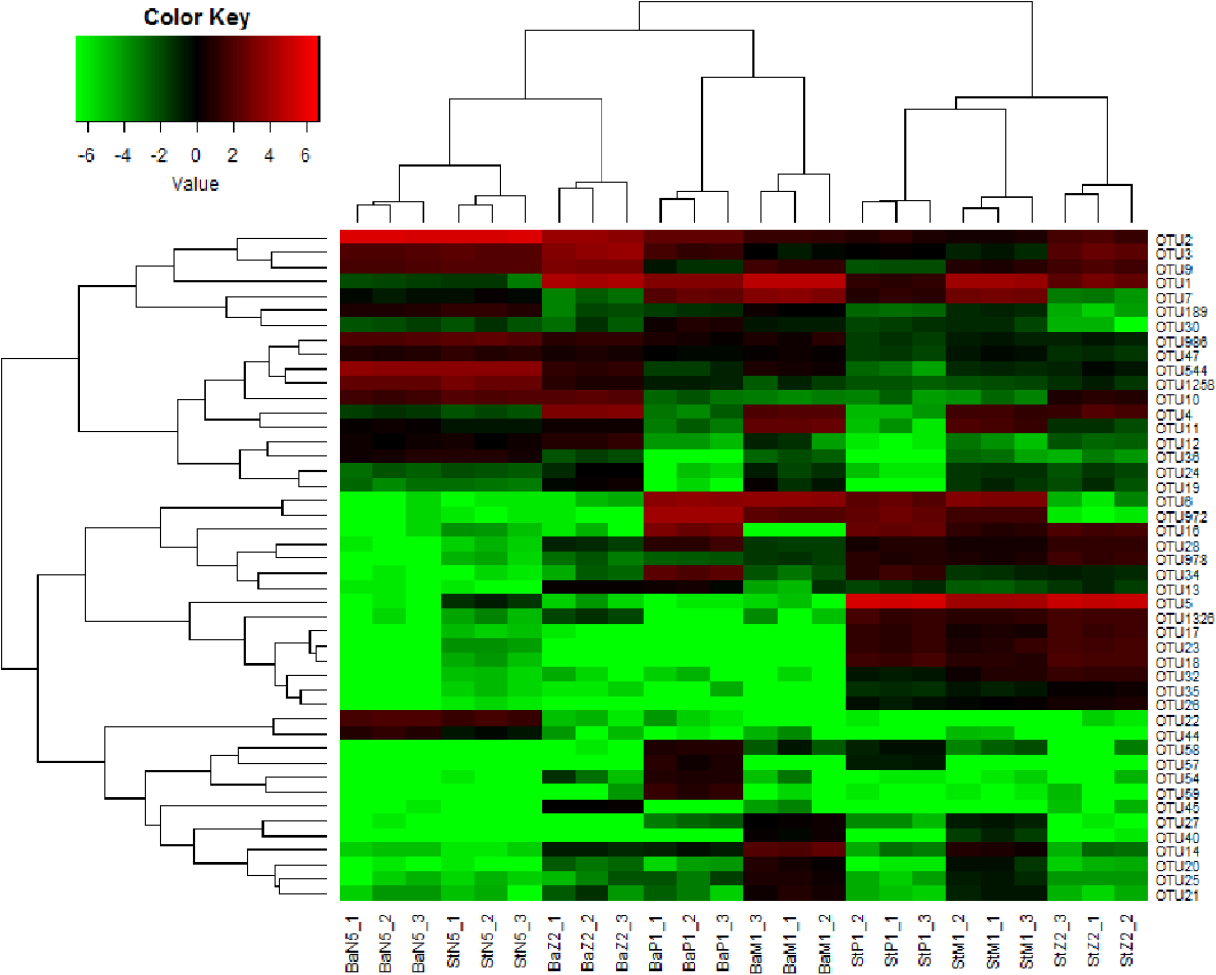
Heat map of cluster relations of kit-based replicates and dominant OTU-based taxa of microbiota associated with *L. vannamei* N5, Z2, M1, and P1 samples, extracted with Ba or St kits. BaN5, BaZ2, BaM1, and BaP1 indicate microbial DNA extracted with the Ba kit from larval samples of N5, Z2, M1, and P1, respectively; numbers 1, 2, and 3 indicate technical replicates of each kit. This is similar for the St kit.

Figure 5 illustrates the cluster relations among the 46 dominant OTUs for larval samples of four stages extracted with the Ba and St kits. Table S3 gives the taxonomical information and relative abundances of these dominant OTUs. Among them, three OTUs, all assigned to Rhodobacteraceae, including OTU2 (*Nautella*/*Aliiroseovarius*), OTU544 (unclassified genus) and OTU1258 (*Loktanella*), predominated at N5 stage. Especially, OTU2 had relative abundance in range of 45.18–50.34%, whereas all three OTUs dropped greatly in abundance at later larval stages. OTU3 (*Donghicola*) and OTU9 (unclassified genus) of Rhodobacteraceae also dominated during the earlier larval period. In contrast, the other five OTUs, including OTU1 (Flavobacteriaceae, unclassified genus), OTU6, OTU972, and OTU7 (Rhodobacteraceae, unclassified genera), and OTU16 (Moraxellaceae, unclassified genus) prevailed at certain later larval stages.

The heat map revealed that each minimum cluster was composed of its own three technical replicates. Except for N5 larvae, other samples clustered by kit utilized, resulting in two main clades. This finding also implied that there were differences between Ba and St extracts. A total of 26, 38, 40, and 39 OTUs were dominant in the microbiota of N5, Z2, M1, and P1, respectively. Among these OTUs, the proportions of those with significant differences between the Ba and St kits were up to 46.2%, 86.8%, 75.0%, and 84.6% (*P* < 0.05). Therefore, the differences in relative abundances of these dominant OTUs further reflected extraction preferences of these two kits.

## Discussion

For detailed microbiota characterization, high-fidelity genomic DNA amplification is vital for 16S rRNA gene amplicon sequencing. In the present study, although larval exoskeletons offer special challenges to DNA extraction, the DNA yields of the four kits were sufficient, since the DNA template requirement can be as low as 5–10 ng (Kennedy et al., 2014a). Additionally, genomic DNA of shrimp larvae may also be extracted by any one of the kits, especially kits Mo and Ti. Nonetheless, the impact of larval DNA on the specific amplification of 16S rDNA might be negligible, given sufficient contents of microbial DNA provided and subsequently strict quality control of sequencing data. Similarly, Guo & Zhang (2013) reported that DNA yield is generally not considered as a screening criterion for extraction methodology. No obvious correlation has been detected between DNA yield and representation of microbial diversity (Salonen et al., 2010; Yuan et al., 2012). Notably, the yield of the St kit was far lower than that of the other three kits. The loss is possibly due to the additional DNA purification with a matrix for inhibitor absorption. Nevertheless, this problem may not affect the microbiota characterization of larval samples, since the St kit resulted in relatively higher Shannon diversity and richness indices. Likewise, Yuan et al. (2012) reported that DNA extraction methods, giving relatively lower DNA yield, represent the true microbial diversity more accurately.

The DNA yield of the Ti kit varied greatly depending on the method chosen for quantification. For example, there was a 3.9-fold greater DNA yield with Nanodrop than with PicoGreen. The NanoDrop method is based on absorbance of components at 260 nm, via which both DNA and non-DNA can be detected, whereas PicoGreen quantification is more reliable because it is specific for double-stranded DNA (Ahn, Costa & Emanuel, 1996). Therefore, this large difference implied that impurities or inhibitors remain in the Ti extracts, thereby affecting downstream procedures and resulting in poor technical reproducibility.

Thus, reproducibility is crucial for screening of DNA extraction methods. In this study, to avoid biological variation in the evaluation of kit performance, identical homogenate of Z2 sample was used for each kit. However, the similarity metrics, i.e., Jaccard, Bray–Curtis, and Sorensen, all differed significantly among the kits, and showed maximum levels within the Ba extracts. These data implied that kit Ba has the best reproducibility, as also verified by the DCA plot. Conversely, the inferior reproducibility of the Ti kit was demonstrated by the separation of three replicates. Thus, the Ti kit was inappropriate for microbial DNA extraction from shrimp larvae.

For Z2 larvae, there was a significant difference in relative abundances of dominant phyla/classes among the four kits. Nevertheless, it is difficult to determine which kit yielded more accurate representation of bacterial profiles. Remarkably, Yuan et al. (2012) reported that bacterial species abundance levels are significantly different from the expected values of a mock community for six DNA extraction methods. Kennedy et al. (2014b) also found significant differences in bacterial composition when different kits are employed to extract DNA from fecal samples. Conversely, Rubin et al. (2014) reported that DNA extraction protocols cause differences in DNA yield and amplification efficiency, but no obvious differences in microbial community composition associated with ants. Herein, although resulting in a microbiota structure similar to that of the Mo kit, the Ba kit was superior to the former in terms of reproducibility.

Currently, there are multiple criteria such as DNA yield, DNA shearing, reproducibility, as well as representativeness, which have been used to evaluate DNA extraction methods (Yuan et al., 2012; Kennedy et al., 2014b). The ability to recover Gram-positive bacteria is also crucial to the selection of an optimal DNA extraction kit (Guo & Zhang, 2013). Notably, Guo & Zhang (2013) suggested that the efficiency of DNA extraction kit is proportional to the amount of Gram-positive bacteria detected. In the current study, the relative proportions of the two Gram-positive phyla, Firmicutes, and Actinobacteria, were 46.76%, 7.67%, 5.38%, and 2.62% corresponding to kits St, Ti, Ba, and Mo for Z2 samples, respectively. These drastic differences among the kits implied that the recovery of Gram-positive bacteria seems to be heavily influenced by extraction methodologies. Because different kit procedures may cause different lysis efficiencies, this phenomenon may introduce bias against accurate microbial community profiling (Carrigg et al., 2007; Inceoglu et al., 2010; Yuan et al., 2012). However, the superior ability of the St kit to recover Gram-positive bacteria is indisputable.

In addition, the St kit was desirable for gaining bacterial assemblages associated with the shrimp intestine, such as the families Streptococcaceae, Leuconostocaceae, and Enterobacteriaceae. This kit apparently showed higher relative abundances than did the Ba kit for samples Z2, M1, and P1. The presence of these bacterial taxa implied that kit St is better at disrupting the larval exoskeleton and tissues within, which may be due to mechanical lysis by glass beads. To date, mechanical disruption by bead-beating is more suitable to lyse certain types of cells in diverse samples. Chemical and enzymatic lysis methods suffer from the extraction bias due to limited spatial access to target organisms (Carrigg et al., 2007; Guo & Zhang, 2013; Kennedy et al., 2014b). Salonen et al. (2010) reported that mechanical cell disruption by repeated bead-beating presents the highest bacterial diversity in fecal samples, and results in significantly better DNA extraction efficiency for archaea and some bacteria. Maukonen, Simões & Saarela (2012) reported that, in comparison with mechanical lysis kits, the number and composition of clostridial groups are significantly lower when enzymatic extraction kits being used. Yuan et al. (2012) also found extraction methods that including bead-beating and mutanolysin could produce significantly better representation of bacterial community structure. However, this was not the case for the Ba kit, although a lengthy vortexing step with glass powder is also included. In contrast, using DNA extraction methods similar to the Ba kit, which include bead-beating with quartz sand and subsequent digestion with lysozyme and proteinase K, Zheng et al. (2017) reported that Enterobacteriaceae accounts for more than 85% of the total bacteria, and was the only overwhelming group at all shrimp larval stages. This large discrepancy with the results of the present study awaits further investigation, though the rearing environment and diet feeding may act as important influencing factors.

For the St kit, the unique step of inhibitor-adsorption with matrix during DNA purification may favor the removal of PCR inhibitors that affect the amplification of gut bacterial DNA. This approach seems possible due to the diverse PCR-inhibiting materials present in the intestine (Nechvatal et al., 2008; Rubin et al., 2014; Larsen, Mohammed & Arias, 2015). Aside from bead-beating and PCR inhibitor removal, thermal shock may also contribute to the recovery of Gram-positive and some intestine-associated bacteria. Likewise, heat treatment had been reported to have good lysis efficiency in other commercial fecal DNA extraction kits (Dridi et al., 2009).

As mentioned above, the Ba kit showed good reproducibility for characterization of larval microbiota. The St kit was complementary with its ability to identify many biologically important taxa. For example, four families, and specifically Streptococcaceae, Leuconostocaceae, Carnobacteriaceae, and Lactobacillaceae, all belonging to lactic acid bacteria (LAB), were present in significantly higher proportions with the St kit than with the Ba kit for the Z2 sample. These dominant bacterial groups, together with Enterobacteriaceae species, are mostly gut-residents. This finding implied that the St kit also has good efficacy of recovery for these specific bacterial groups, with the exception of stage N5, which has not begun feeding on exogenous food. In particular, OTU5 (Streptococcaceae, *Lactococcus*), as the second most dominant group with the St kit, showed high proportions of 33.28%, 17.87%, and 42.45% at larval stages Z2, M1, and P1, respectively. This dominance indicated potential probiotic effects of OTU5 on shrimp larvae, because many LAB species are thought to be beneficial microbes for aquatic animals.

It is important to stipulate that all samples in an experiment should be extracted by the same methodology. This is because differences in DNA extraction protocols may bias results and complicate inter-study comparisons (Pérez-Losada, Crandall & Freishtat, 2016). For some biological or environmental samples without background information, the combination of multiple methodologies will be of particular interest to access the uncultured majority of microbes. For instance, in this study, the advantage of the St kit was its ability to recover gut-associated bacteria, mainly from Firmicutes, and to facilitate identification of indigenous probiotic bacteria. Actinobacteria, a subdominant Gram-positive group, appeared with relatively higher proportion with the Ba kit, allowing for the detection of more GC-rich taxa.

It is well known that the most common bacteria pathogenic to shrimp larvae are vibrios. The relative abundances of *Vibrio*, showed higher proportions with the Ba kit (ranged in 0.02–1.75%) than with the St kit (0–0.66%) regardless of larval stages. In addition, OTU2 was phylogenetically affiliated with genera *Nautella* and/or *Aliiroseovarius* and manifested high but different relative abundance levels with kits Ba and St. OTU2 yielded proportions of 49.24%, 13.97%, 2.53%, and 5.44% with the former kit as compared to 46.27%, 3.29%, 1.51%, and 1.90% with the latter for larval samples N5, Z1, M1, and P1, respectively. *Nautella* had been reported to be associated with diseased larvae of *L. vannamei* (Zheng et al., 2016, 2017) and might be opportunistic pathogenic bacteria for shrimp larvae. *Aliiroseovarius crassostreae* was reported as the causative agent of Roseovarius oyster disease (Kessner et al., 2016). This result indicated that shrimp larvae, especially nauplius, could enrich *Nautella*/*Aliiroseovarius* (opportunistic pathogens). Moreover, the Ba kit will be more effective at DNA isolation from *Vibrio* and *Nautella*/*Aliiroseovarius* associated with shrimp larvae, because this kit always yielded higher proportions of these potential pathogens.

## Conclusions

Overall, the bacterial community of *L. vannamei* larvae was found to be highly dynamic throughout larval development, with phyla Proteobacteria, Bacteroidetes, and Firmicutes, and families Rhodobacteraceae, Flavobacteriaceae, Streptococcaceae, and Moraxellaceae as the most abundant groups during the larviculture period. The biases in shrimp larval microbiota structure may be mainly ascribed to the cell lysis and DNA purification by the four kits used for DNA extraction. Technical reproducibility and capacity for recovery of Gram-positive bacteria are two key factors for choosing a DNA extraction kit. The Ba and St kits are recommended for achieving a fair representation of larval microbiota because these kits are complementary in identifying biologically important groups. Therefore, a combination of multiple protocols should receive adequate attention for effective elucidation of the larvae-associated microbiota of penaeid shrimp.

## Supplemental Information

10.7717/peerj.5257/supp-1 Supplemental Information 1 Table S1. Summary of the sequencing depth for each sample. The bold numbers of 37,722 and 18,180 were used as the resampling levels for Z2 samples with four kits, and for four larval-stage samples with Ba and St kits, respectively, since they were the lowest sequencing depths for the respective samples.

10.7717/peerj.5257/supp-2 Supplemental Information 2 Table S2. Dominant bacterial families associated with Z2 larvae extracted with four kits. Means with different superscripts in the same row are significantly different (*P* < 0.05, *n* = 3).

10.7717/peerj.5257/supp-3 Supplemental Information 3 Table S3. Taxonomical information and sequence abundance of dominant OTUs. BaN5, BaZ2, BaM1, and BaP1 indicate microbial DNA extracted from larval samples of N5, Z2, M1, and P1 with the Ba kit, respectively; Numbers 1, 2, and 3 indicate technical replicates of each kit. This is similar for the St kit.

## Additional Information and Declarations

### Data Availability

The following information was supplied regarding data availability: Raw data is deposited in the Genome Sequence Archive in BIG Data Center, http://bigd.big.ac.cn/, under accession number CRA000198.
