# Supplementary material for: Biases during DNA extraction affect characterization of the microbiota associated with larvae of the Pacific white shrimp, Litopenaeus vannamei
Source: PeerJ. 2018 Jul 16;6:e5257. doi: 10.7717/peerj.5257 (PMC6052851; doi:10.7717/peerj.5257)
Supplement: Supplemental Information 1 — The bold numbers of 37,722 and 18,180 were used as the resampling levels for Z2 samples with four kits, and for four larval-stage samples with Ba and St kits, respectively, since they were the lowest sequencing depths for the respective samples. [file peerj-06-5257-s001.docx]

| Sequencing run | Sample ID | Sequencing depth | |
| --- | --- | --- | --- |
|  |  | After quality control | After removing eukaryotes |
| First | BaZ2_1 | 54,814 | 54,462 |
|  | BaZ2_2 | 90,310 | 90,026 |
|  | BaZ2_3 | 58,581 | 58,423 |
|  | MoZ2_1 | 58,032 | 57,710 |
|  | MoZ2_2 | 55,072 | 54,908 |
|  | MoZ2_3 | 55,592 | 55,291 |
|  | StZ2_1 | 48,153 | 48,127 |
|  | StZ2_2 | 46,047 | 46,009 |
|  | **StZ2_3** | 37,769 | **37,722** |
|  | TiZ2_1 | 56,622 | 56,516 |
|  | TiZ2_2 | 47,860 | 47,809 |
|  | TiZ2_3 | 46,195 | 46,039 |
| Second | BaN5_1 | 20,449 | 20,444 |
|  | BaN5_2 | 28,298 | 28,284 |
|  | BaN5_3 | 22,289 | 22,285 |
|  | BaM1_1 | 70,419 | 70,386 |
|  | BaM1_2 | 62,832 | 62,819 |
|  | BaM1_3 | 121,240 | 121,062 |
|  | BaP1_1 | 19,628 | 19,623 |
|  | BaP1_2 | 19,168 | 19,168 |
|  | **BaP1_3** | 18,182 | **18,180** |
|  | StN5_1 | 26,864 | 26,863 |
|  | StN5_2 | 29,756 | 29,755 |
|  | StN5_3 | 26,750 | 26,747 |
|  | StM1_1 | 56,653 | 56,651 |
|  | StM1_2 | 54,629 | 54,624 |
|  | StM1_3 | 54,842 | 54,823 |
|  | StP1_1 | 18,824 | 18,824 |
|  | StP1_2 | 19,035 | 19,030 |
|  | StP1_3 | 21,187 | 21,187 |
