# Supplementary material for: Biases during DNA extraction affect characterization of the microbiota associated with larvae of the Pacific white shrimp, Litopenaeus vannamei
Source: PeerJ. 2018 Jul 16;6:e5257. doi: 10.7717/peerj.5257 (PMC6052851; doi:10.7717/peerj.5257)
Supplement: Supplemental Information 2 — Means with different superscripts in the same row are significantly different (P < 0.05, n = 3). [file peerj-06-5257-s002.docx]

| Phylum / Class | Family | Kits | | | |
| --- | --- | --- | --- | --- | --- |
|  |  | Ba | Mo | St | Ti |
| α-Proteobacteria | Rhodobacteraceae | 48.49±0.72 ^a^ | 46.35±3.95 ^a^ | 15.97±2.46 ^c^ | 31.25±2.46 ^b^ |
| β-Proteobacteria | Comamonadaceae | 0.50±0.03 ^bc^ | 1.34±0.41 ^b^ | 0.18±0.01 ^c^ | 26.98±3.87 ^a^ |
|  | Oxalobacteraceae | 0.09±0.06 ^b^ | 0.22±0.31 ^b^ | 1.13±0.11 ^a^ | 0.47±0.35 ^b^ |
| γ-Proteobacteria | Alteromonadaceae | 1.90±0.180 ^a^ | 1.64±0.08 ^ab^ | 0.66±0.09 ^c^ | 1.35±0.32 ^b^ |
|  | Enterobacteriaceae | 0.29±0.08 ^b^ | 0.34±0.08 ^b^ | 2.83±0.24 ^a^ | 0.25±0.13 ^b^ |
|  | Moraxellaceae | 2.28±0.22 ^b^ | 4.21±2.17 ^ab^ | 12.46±0.63 ^a^ | 9.03±7.51 ^ab^ |
|  | OTU_19 (Unclassified) | 1.11±0.16 ^a^ | 0.94±0.29 ^a^ | 0.35±0.11 ^b^ | 0.09±0.08 ^c^ |
| Firmicutes | Bacillaceae | 1.18±0.03 ^a^ | 0.81±1.19 ^ab^ | 0.17±0.21 ^b^ | 0.77±0.27 ^ab^ |
|  | Listeriaceae | 0 ^b^ | 0 ^b^ | 2.89±0.35 ^a^ | 0 ^b^ |
|  | Leuconostocaceae | 0.04±0.02 ^b^ | 0 ^c^ | 3.75±0.33 ^a^ | 0.01±0.01 ^c^ |
|  | Streptococcaceae | 1.03±0.31 ^b^ | 0.06±0.04 ^c^ | 38.04±2.82 ^a^ | 0.29±0.02 ^c^ |
| Bacteroidetes | Flavobacteriaceae | 22.35±1.60 ^a^ | 24.25±2.97 ^a^ | 8.70±1.15 ^b^ | 9.60±1.05 ^b^ |
|  | Cyclobacteriaceae | 9.53±0.64 ^a^ | 10.15±0.84 ^a^ | 3.45±0.9 ^c^ | 5.26±1.23 ^b^ |
| Actinobacteria | Nocardioidaceae | 1.99±0.34 ^b^ | 0.39±0.07 ^c^ | 0.17±0.06 ^d^ | 5.22±0.51 ^a^ |
